# Epidemic interventions: insights from classic results

**DOI:** 10.1098/rstb.2020.0263

**Published:** 2021-07-19

**Authors:** Julia R. Gog, T. Déirdre Hollingsworth

**Affiliations:** ^1^ Department of Applied Mathematics and Theoretical Physics, University of Cambridge, Cambridge CB3 0WA, UK; ^2^ Big Data Institute, Li Ka Shing Centre for Health Information and Discovery, University of Oxford, Oxford OX3 7LF, UK

**Keywords:** epidemic, pandemic, non-pharmacutical interventions, SIR model

## Abstract

Analytical expressions and approximations from simple models have performed a pivotal role in our understanding of infectious disease epidemiology. During the current COVID-19 pandemic, while there has been proliferation of increasingly complex models, still the most basic models have provided the core framework for our thinking and interpreting policy decisions. Here, classic results are presented that give insights into both the role of transmission-reducing interventions (such as social distancing) in controlling an emerging epidemic, and also what would happen if insufficient control is applied. Though these are simple results from the most basic of epidemic models, they give valuable benchmarks for comparison with the outputs of more complex modelling approaches.

This article is part of the theme issue ‘Modelling that shaped the early COVID-19 pandemic response in the UK’.

## 1. Introduction

During the first wave of the COVID-19 pandemic, many analyses were made on the potential impact of the pandemic and possible mitigation measures, for example reports considered by SAGE (the UK government’s Scientific Advisory Group for Emergencies) in February 2020 ([1]). Many of these analyses depended on intricate models which take into account some of the complexity of transmission of COVID-19 in the UK human population ([1] and subsequently [2]). While so many of these complexities were potentially of importance for predicting the likely future trajectory of the pandemic, elaborate models—and the consequent strong dependence on the details of computational implementation—are open to criticism regarding the robustness of their results. At the other end of the spectrum, the simplest models can be used to give broad insights and will be robust to implementation, and the dependence on assumptions is transparent. While the simplest models are rarely appropriate for precise projections, they can give valuable benchmarks against which results from more complex models can be evaluated: when they are broadly similar both qualitatively and quantitatively this gives confidence that shared results are robust, and when they differ that may highlight some elements of the more intricate models having substantial effect in the context considered.

In this paper, we expand on some classic results which were presented to SAGE in February 2020 (Gog [3]) which in turn drew on Hollingsworth *et al.* ([4]). Analytic results can be obtained from the classic SIR (susceptible-infected-removed) model which depend only on the single epidemiological parameter *R*_0_. Here, we consider the impact of interventions which have the effect of reducing transmission (called NPIs—non-pharmaceutical interventions—for example ‘lockdowns’). The interventions themselves are modelled here in minimal complexity, for example that the effect on transmission starts instantly and is constant in time. Early in COVID-19, the focus was on assessing what might be ahead of us, to understand what levels of intervention were necessary to bring under control both the peak prevalence (thinking of critical care capacity) and also the total number of people infected over the epidemic (thinking of cumulative impact of illness and number of deaths). We consider both of these measures below.

In this paper, we aim to provide accessible analytical results for two key policy questions, which were posed at various stages of the COVID-19 pandemic in the UK:

— In the early stages of a new epidemic, what is the effect of some kind of social distancing or ‘lockdown’ on the dynamics? Can we wait before introducing interventions, if so how long should we wait? What does this mean for how ‘harsh’ the lockdown has to be?
— If we introduce a lockdown when there is partial immunity in the population, how much does this change the dynamics?

We first present classic analytic results from the classic SIR model which give baseline results for an unmitigated epidemic and set up the mathematics needed for subsequent analyses. Next, we consider the effect of interventions that are applied part way into the outbreak. Finally, we consider the effect of interventions in the context of partial population immunity, arising through natural immunity through infection or through vaccination.

## 2. Classic results without intervention

The classic model for an outbreak is described by the SIR system. Here, we just need the equations for *S*(*t*), the proportion of the population susceptible at time *t*, and *I*(*t*), the proportion of the population infected and infectious at time *t*. Rescaling time so that the infectious period has mean duration 1, the system is given by:

$$\dot{S}=-rSI$$

$$\dot{I}=+rSI-I,$$

where *r* here denotes the basic reproduction ratio (*R*_0_). This basic but elegant model includes many simplifying assumptions, particularly no latent period, exponential distribution of infectious period, homogeneous mixing and enduring immunity following infection. However, while some simple assumptions are clearly ‘wrong’ for COVID-19, the SIR system provides a parsimonious approximation: a benchmark to which the effects of more elaborate models can be compared. This is particularly true during the early, exponential growth phase of an epidemic.

The crux of the approach taken here is to sidestep the time-dependence. From the above equations, the function *F*(*S*, *I*) is constant in time

2.1 $$F(S,I)=S+I-r^{-1}\log S.$$

Although this no longer captures the full-time evolution of the system, when combined with suitable boundary conditions this result is enough to give both the peak prevalence and also the final size of the epidemic.

For the case without intervention, initially nearly everyone is susceptible, and a very small number of infecteds are introduced, i.e. (*S*, *I*) ≈ (1, 0), thus solutions are close to

F(S,I)=F(1,0)=1.

The peak prevalence is the maximum value of *I* over time, which is also the maximum value of *I* as *S* is varied. Differentiating equation (2.1) with respect to *S* on constant *F*, we see this must be achieved at *S* = *r*^−1^ (for *r* ≥ 1 only, for *r* < 1 there is no epidemic and peak prevalence is zero). Hence solving *F*(*r*^−1^, *I*_max_) = 1 for peak prevalence *I*_max_:

$$I_{\max}=1-\frac{\left(1+\log r\right)}{r}.$$

For final size, the classic approach is to seek the solution at the end of the epidemic, for *S*_∞_ ≠ 1 when *I* = 0. We know from consideration of the initial conditions that *F*(*S*, *I*) = *F*(1, 0) = 1, and, since *F*(*S*, *I*) is constant, then *F*(*S*_∞_, 0) = 1 and so:

$$1=S_{\infty }-r^{-1}\log S_{\infty }.$$

For *r* ≤ 1, there is no real solution with *S*_∞_ < 1 (no epidemic can happen, in which case final size is zero). For *r* > 1, the solution for *S*_∞_ can be written as

$$S_{\infty }=-W[-r\;e^{-r}]/r,$$

where *W* is the Lambert W-function (or product logarithm). The so-called final size is the proportion of population infected at any time during the epidemic, which for the SIR model is one minus those left still susceptible, and here *r* = *R*_0_, hence the final size (FS) is given by

$$\mathrm{FS}=1-S_{\infty }=1+W[-R_{0}e^{-R_{0}}]/R_{0}.$$

**Figure 1.**
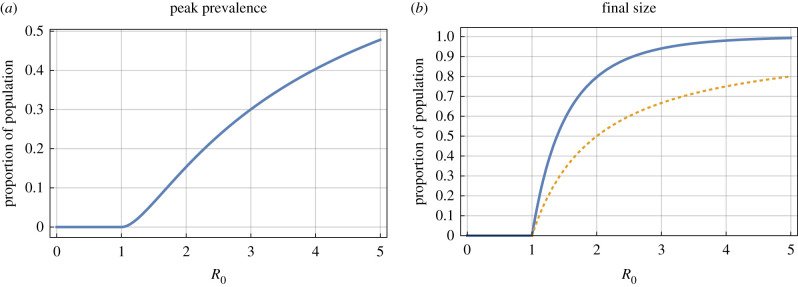
Baseline peak prevalence and final size, as functions of the reproduction ratio, *R*_0_. Peak prevalence (*a*) and final size (*b*) against reproduction ratio *R*_0_, both given as a proportion of the population. The dotted line on the final size plot gives the susceptible proportion when peak prevalance is achieved, which is also the herd immunity threshold. (Online version in colour.)

These classic results for an unmitigated epidemic are plotted in figure 1.

## 3. Effects of intervention

If interventions are brought in during the epidemic, we can still make some headway with informative analytical expressions but as before, again under particular simplifying assumptions about the form and effect of the intervention. Again, these can serve as approximations and benchmarks for more realistic models of intervention. We assume that the intervention has the effect of reducing the reproduction ratio by a proportion *ϕ* (so now *r* = (1 − *ϕ*)*R*_0_). This could be through a reduction in transmission rates, or shortening of duration of infectiousness. To take full advantage of the time-independence of the above results, assume that the intervention is applied for long duration (mathematically, effectively through to the end of the epidemic). We identify the time that the intervention is started by there being a proportion *θ* that has been infected, i.e. $S_{*}=1-\theta$. It could be that this itself is the description of control strategy: apply intervention when cumulative incidence reaches this trigger threshold. Or it could be that there is some other extrinsic trigger, but the effect is that intervention is applied at this point in the epidemic.

With this form of intervention, rather than just matching from initial to final state of the epidemic, we match in two separate sections: from initial to start of intervention, and then start of intervention to final. For the first period, before intervention, *r* = *R*_0_. We take again (*S*, *I*) ≈ (1, 0) initially, and at time that the intervention begins, we have $\left(S,I)=(S_{*},I_{*}\right)$ and we find $I_{*}$ using $F(1,0)=F(S_{*},I_{*})$ with equation (2.1) and $S_{*}=1-\theta$. Solving for $I_{*}$:

$$I_{*}=\theta +R_{0}^{-1}\log(1-\theta ).$$

For the second phase, from start of intervention to end of epidemic, we find *I*_∞_ using $F(S_{*},I_{*})=F(S_{\infty },0)$, this time with $r=r_{*}=(1-\phi )R_{0}$:

$$r_{*}(1-S_{\infty })+\log S_{\infty }=\phi \log S_{*}.$$

Again, we can write the solution using a W-function:

$$S_{\infty }=-W[-r_{*}S_{*}^{\phi }e^{-r_{*}}]/r_{*},$$

hence now the final size, written out in full, is given by

$$\mathrm{FS}=1-S_{\infty }=1+\frac{W[-(1-\phi )R_{0}\;{\left(1-\theta \right)}^{\phi }e^{-(1-\phi )R_{0}}]}{(1-\phi )R_{0}}.$$

It is useful to look at this relative to the unmitigated final size, so that we can give a sense of how much a given intervention would reduce epidemic size. The relative final size is simply the final size of a mitigated epidemic divided by the original final size (given above):

$$\mathrm{Relative}\;\mathrm{FS}=\frac{R_{0}+W[-(1-\phi )R_{0}\;{\left(1-\theta \right)}^{\phi }e^{-(1-\phi )R_{0}}]/(1-\phi )}{R_{0}+W[-R_{0}e^{-R_{0}}]}$$

Some care is needed when calculating the peak prevalence under intervention as there are multiple cases as to when the peak occurs. If the intervention is applied very late, the peak will already have occurred as per the unmitigated case. If the intervention is initiated before the original peak but is a weak intervention, the peak will be in the future during the mitigated phase. If the mitigation is applied early and is strong, then the epidemic can immediately turn from growing to shrinking and then the peak prevalence is the time when intervention begins (i.e. $I_{\max}=I_{*}$). The solutions for peak prevalence thus follow analogously to above, but with care over these cases:

$$I_{\max}=\left\{ \begin{array}{ll} 1-\frac{\left(1+\log r_{*}+\phi \log(1-\theta )\right)}{r_{*}} & \mathrm{if}\;\theta <1-\frac{1}{r_{*}}\\ \theta +\frac{\left(\log(1-\theta )\right)}{R_{0}} & \mathrm{if}\;1-\frac{1}{r_{*}}<\theta <1-\frac{1}{R_{0}}\\ 1-\frac{\left(1+\log R_{0}\right)}{R_{0}} & \mathrm{if}\;1-\frac{1}{R_{0}}<\theta \end{array}\right.$$

where $r_{*}=(1-\phi )R_{0}$. Note that if the intervention is strong enough that $r_{*}<1$, the first condition above cannot occur. Then again just take the appropriate ratio with the peak prevalence without intervention to find the relative peak prevalence.

$$\begin{array}{ll} & \mathrm{relative}\;I_{\max}\\ & \;=\left\{ \begin{array}{ll} \frac{R_{0}-(1+\log[R_{0}(1-\phi )]+\phi \log(1-\theta ))/(1-\phi )}{R_{0}-1-\log R_{0}} & \mathrm{if}\;\theta <1-\frac{1}{r_{*}}\\ \frac{\theta R_{0}+\log(1-\theta )}{R_{0}-1-\log R_{0}} & \mathrm{if}\;1-\frac{1}{r_{*}}<\theta <1-\frac{1}{R_{0}}\\ 1 & \mathrm{if}\;1-\frac{1}{R_{0}}<\theta \end{array}\right. \end{array}$$

**Figure 2.**
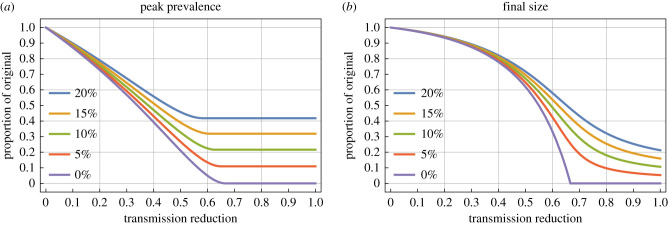
Effect of intervention, by strength of intervention. The relative peak prevalence (*a*) and relative final size (*b*) as function of the transmission reduction of the intervention (*ϕ*) for *R*_0_ = 3. In each, the different coloured lines represent different trigger values for application of the intervention *θ*.

**Figure 3.**
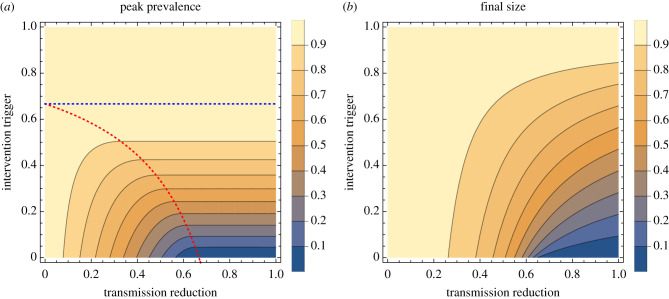
Effect of intervention, by strength and timing of intervention. The relative peak prevalence (*a*) and relative final size (*b*) as function of both the transmission reduction of the intervention (*ϕ*) and trigger for application of the intervention (*θ*) for *R* = 3. For the peak prevalence plot (*a*), the additional dotted red and blue lines separate the different cases for the timing of peak incidence relative to intervention timing: below the red line, cases are rising at the time intervention is applied and the intervention is not strong enough to immediately turn over the epidemic; between the red and blue lines, the intervention is enough to immediately turn over the epidemic (and hence the contours are horizontal in this region); above the blue dotted line, the epidemic has already peaked before intervention is applied, so the intervention does not affect the peak prevalence.

These results are illustrated in figures 2 and 3 for an *R*_0_ of 3.

## 4. Results in the context of COVID-19 in early 2020

For the illustrative plots presented above, we have used a value of the reproduction ratio *R*_0_ = 3. This is a higher value than the *R*_0_ = 2.2 used in the analysis which was presented to policy in February 2020 [3]. That value of *R*_0_ was matched to that used in the Imperial College paper which was submitted to the same meeting of SAGE [1]. Davies *et al.* performed a meta-analysis of available studies and estimated that *R*_0_ was 2.7 (95% credible interval 1.6–3.9) and the Imperial College analysis used 2.2 as a base value (driven by a 5 day doubling time), but investigated values from 2 to 2.4 (their subsequent report used 2 to 2.6) [1,5,6]. A subsequent systematic review and meta-analysis, published in November 2020, gave a summary estimate of 2.87 (95% CI, 2.39–3.44) [7]. Therefore, the value used here, *R*_0_ = 3, is at the higher range of values used at the time, but in keeping with both original and current estimates.

Inspection of the analytic results demonstrates there will be no qualitative changes in using different *R*_0_ (so long as *R*_0_ > 1), but there will be qualitative changes corresponding to a change in *R*_0_. For figure 2, the effect of a higher reproduction ratio would be to require a stronger transmission reduction for the same effect sizes—the plot is qualitatively the same but the lines will move mainly to the right.

**Figure 4.**
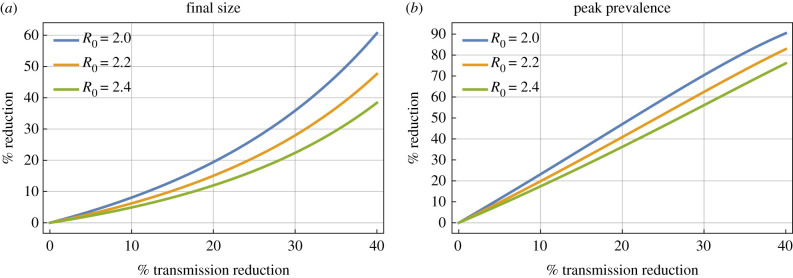
Effects of intervention, matched to values seen in SAGE meeting 10. The percentage reduction in final size (*a*) and peak prevalence (*b*) as function of the transmission reduction of the intervention (now also as percentage) for *R* = 2.0, 2.2 and 2.4, chosen for comparison to table 1 in Imperial College paper [1].

For the purposes of historic comparison, and also to show how this simple approach can be used in practice as a benchmark for outputs from other models, figure 4 gives the classic results for *R* = 2.0, 2.2 and 2.4 and the *y*-axis is given in terms of percent reduction, to aid a side-by-side comparison with table 1 of the Imperial College analysis [1]. Final size is not exactly the same as the reduction in attack rate over 26 weeks, but if the main wave of the epidemic is mostly over by that time, then it will be a fair comparison. Similarly, prevalence and incidence are of course different, but their proportional reduction in peak should be comparable for an acute infection.

As specific example, in the first row of table 1 of the Imperial College report [1], intervention ‘PC’ (closure of schools and universities) is reported to give a reduction in attack rate of 11%, 8% and 6% for the three values of *R*_0_. Reading off figure 4, this would correspond in the simpler model to a transmission reduction of around 10–15%. This is broadly consistent with the range required for the reported peak incidence, though the values do not exactly match up, suggesting that some additional factor in the Imperial College analysis [1] means that the effects on overall attack rate are weaker, or on peak incidence are stronger, than that expected in the model presented here. A key difference is that in our analysis NPIs applied indefinitely whereas for the Imperial College analysis they are for 13 weeks only [1]. However, if this is the crux of any differences, then this raises questions about the necessary duration of interventions and what happens after they are lifted.

Finally, figure 4 allows the reader to see that results in any model are likely to be highly sensitive to the assumptions about strength of transmission reduction, and, in particular in the range here, any strengthening of transmission reduction would have a better than linear effect on the final size (the lines in figure 4 curve upwards). This is more immediate than rerunning many simulations of a complex model, and makes robust insights available to non-specialists.

## 5. Intervening in the presence of population immunity

**Figure 5.**
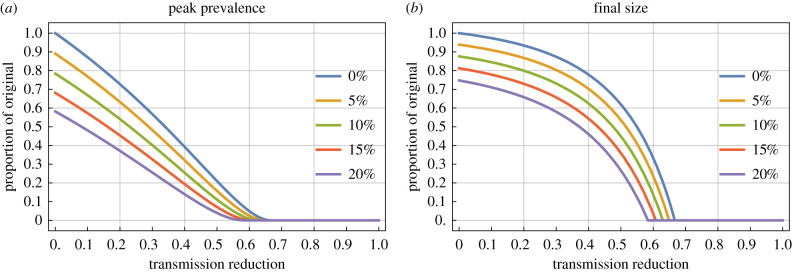
Effect of prior immunity, by strength of intervention. The relative peak prevalence (*a*) and relative final size (*b*) as function of the transmission reduction of the intervention (*ϕ*) for *R* = 3, assuming intervention applied as soon as the epidemic starts. In each, the different coloured lines represent different levels of prior immunity.

**Figure 6.**
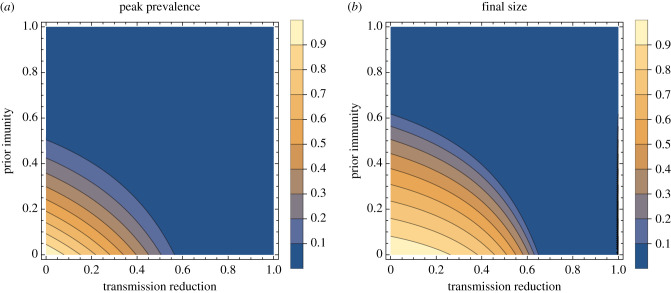
Effect of intervention, by strength of intervention and level of prior immunity. The relative peak prevalence (*a*) and relative final size (*b*) as function of both the transmission reduction of the intervention (*ϕ*) and level of prior immunity for *R* = 3, assuming interventions are applied as soon as the epidemic starts. Here, interventions are applied quickly, so they entirely stop any subsequent epidemics if they are strong enough. Some prior immunity means that a lower strength of intervention will be sufficient to entirely stop a subsequent outbreak. Even an imperfect intervention may be enough to dramatically reduce the outbreak with a modest level of prior immunity.

As the pandemic is progressing, the question arises to what extent the predicted effects of intervention are changed if there is some immunity in the population, either through natural infection or vaccination. Here, intervention starts early (*θ* = 0) but there is some prior immunity. Mathematically, this follows on easily from the above results, just using more general initial conditions: *S*(0) = *S*_0_, and note now that final size is *S*_0_ − *S*_∞_. Also, care is needed for both peak and final size calculations to check that effective reproduction ratio, *R*, or the number of transmissions per infected individual at this point in the epidemic, is greater than 1 initially [8,9]. In this context, this means that the epidemic growth rate is positive (i.e. the epidemic is growing) at the time that the interventions are introduced. The results are shown in figures 5 and 6.

The results here are intuitive, given the classic threshold results for epidemics: if there is some prior immunity, then even a previously weak intervention might be enough to greatly reduce or even prevent the epidemic. The crux is whether or not effective *R* is greater or less than 1. And if only just greater, the peak prevalence and final size could be small.

These results are qualitatively robust to different assumptions on how the immunity has come about, but we know that different dynamics can arise depending on the duration of immunity [10], and how acquisition of immunity is correlated with transmission [11]. The sensitivity to the reproduction ratio is similar to that described in §4, above.

## 6. Discussion and conclusion

Transmission reduction by non-pharmaceutical interventions can be captured in the simplest epidemic models, giving a ballpark estimate for the size of the effect both in terms of reduction of overall attack rate and peak incidence. There are clear insights gained from these classic analyses' simple models: (a) effective, long-term interventions must be both strong enough (to bring *R* < 1) and applied early (figure 3) to have minimal total cases and peak prevalence; (b) in the presence of pre-existing population immunity, less stringent interventions are needed (figure 6); and (c) if an intervention is both applied early and is strong enough to reduce the effective reproduction ratio to below 1, then there is diminishing additional benefit from using an even stronger intervention (figures 2 and 5). These insights are intuitive to disease modellers, but perhaps they have only become intuitive as the field of infectious disease modelling is built upon models such as these. The outputs of these parsimonious models can give more though: for example they show the mathematical interdependence between intervention strength, timing and host immunity on the dynamics of the system—insights which lie beyond just the direction of dependence, as the nonlinear results in §5 demonstrate.

For detailed application for policy design, clearly more elaborate models would be appropriate to give more precise quantitative estimates. Perhaps the most immediate necessary extension from the classic SIR model to COVID-19 is population heterogeneity, both in terms of contact and mixing patterns (age, geography, households, context) and in terms of biological susceptibility and infectiousness, plus many other considerations such as vulnerability to disease by age and other factors. Much of the explosion of modelling preprints in 2020 focused on these host heterogeneities, for example reporting that ‘herd immunity’ can be achieved with fewer people immune than that predicted by homogeneous mixing (‘the most rediscovered result in epidemiology’, V. Andreasen 2020, personal communication). However, insights are still possible to be gained from mathematical approaches (e.g. [12]).

Even with far more complex models with parameter uncertainty, we advocate a role still for simplified models as benchmarks. For example, from a complex model, a prediction is made that a social distancing strategy could reduce overall attack rate by 23% for *R*_0_ = 2.2 ([1], example entry in first table); then a benchmark comparison with classic model results would confirm that this is consistent with a measure that caused a net reduction to transmission of about 30% with interventions applied early ([3], reading off top figure). While the simple models are clearly not a replacement for detailed simulations from complex models, they can thus give ‘rule of thumb’ checks which can either lend confidence to results, or reveal some particular dependence on the assumptions of the more complex model.

In summary, simple models continue to yield valuable insights and can be used alongside more complex models. Perhaps ideal would be a nested approach: a series of models and outputs ranging from the complex and simplifying step-wise towards a fully tractable model. However, such an approach is unlikely to be practical under time pressure. In this case, we advocate a pragmatic approach of at least having the simplest model as a benchmark to compare and contrast. This partnership has the potential to bring in the best of all worlds.

## 7. In context

By late February 2020, it was apparent that some interventions would be required to slow the spread of COVID-19 in the UK and elsewhere. Mathematical models were an essential tool in the policy discussion of when interventions would be needed, how much interventions would be needed, and how long they would have to be held in place.

The UK was fortunate in having a number of world-leading modelling groups providing advice to the government, mainly through the Scientific Pandemic Influenza Group on Modelling (SPI-M). Imperial College London, and the London School of Hygiene and Tropical Medicine provided detailed predictions on the dynamics of the epidemic for different impact and timing of interventions, but comparisons between predictions were difficult due to different assumptions on the dynamics of the epidemic [1,13,14]. In addition, historic modelling suggested that if you could only intervene for a short period of time, then minimizing peak prevalence (to avoid overwhelming hospital capacity) might be best achieved by initiating interventions a little later [4].

In parallel, there was a debate emerging about key parameters, such as the doubling time of the epidemic, and the resulting estimates of the reproduction number, with the Imperial College model being driven by a doubling time of 5 days, giving a reproduction ratio of 2.2, and theManchester University researchers suggesting that doubling times in Europe were closer to 3 days, suggesting a higher reproduction ratio and that action was more urgently needed.

In this context, there was a need for simple approximations and clarity of presentation in the general dynamics of the system. This led to the analysis which was submitted to the Scientific Group for Emergencies (SAGE) upon which this paper is based [3]. Later in the year the need for additional lockdowns, this time in the presence of immunity required additional consideration and some detailed modelling [10], and once again the approximations in this article helped communicate the dynamics of the epidemic in response to such interventions.

The recent history of mathematical epidemiology has been the joint evolution of both complex models and simple approximations which balance each other by providing insight, validation and rigorous comparisons. The mathematics in this article is neither groundbreaking nor novel, but it partners with a wealth of the literature to provide a simplifying framework from which to explain key insights on the impact of NPIs on an emerging, or re-emerging epidemic.

## Data accessibility

This article has no additional data.
